# Pre-service teachers' positive emotions enhance learning of students with low prior knowledge

**DOI:** 10.3389/fpsyg.2026.1914388

**Published:** 2026-08-20

**Authors:** Jie Li, Qian Zhong, Jingjing Guo, Yang Liu

**Affiliations:** 1 Shaanxi Key Laboratory of Behavior and Cognitive Neuroscience, School of Psychology, Shaanxi Normal University, Xi'an, Shaanxi, China; 2 Innovation and Entrepreneurship College, Henan Medical University, Xinxiang, Henan, China; 3 School of Psychology, Henan Medical University, Xinxiang, Henan, China

**Keywords:** interpersonal brain synchronization, learning performance, motivation, pre-service teacher emotion, prior knowledge

## Abstract

**Introduction:**

Recent studies have suggested that teachers' emotions and emotional expressions play an important role in shaping students' learning experiences. However, existing research has primarily relied on self-reported measures, providing limited direct evidence regarding how pre-service teachers' emotional expressions influence learning performance and the underlying interpersonal processes during instruction.

**Method:**

This study examined whether students' prior knowledge moderated the eects of pre-service teachers' positive emotional expressions during face-to-face instruction. Using a dyadic fNIRS approach, it examined students' learning performance, interpersonal brain synchronization.

**Results and discussion:**

The findings revealed an expertise reversal eect: students with lower prior knowledge demonstrated improved learning performance, increased IBS in temporal regions, more positive emotional experiences, and higher learning motivation when exposed to pre-service teachers' positive rather than neutral emotional expressions. In contrast, students with higher prior knowledge showed limited benefits from pre-service teachers' positive emotional expressions. These findings extend the expertise reversal eect to the domain of teachers' emotional expressions, while highlighting the importance of considering learner characteristics when examining emotionally supportive instruction.

## 1 Introduction

Imagine you are learning a second language. Would you prefer a teacher who frequently displays positive emotions? Research on interpersonal interaction indicates that students tend to prefer teachers who exhibit positive emotions. Such emotions can elicit positive responses from students and boost their learning motivation (Bross et al., 2026; Pfeifer et al., 2026; Wang et al., 2026). Prior research has underscored the significant impact of teacher emotions on student learning, motivation, and the enhancement of interpersonal interactions between teachers and students (Frenzel et al., 2021; Wang et al., 2026).

First, teachers' emotions play a pivotal role in shaping the quality of teacher-student interactions (Burić and Frenzel, 2023; Frenzel et al., 2021; Holzer et al., 2024; Xie and Derakhshan, 2021). Positive emotions are particularly important in facilitating social connection and maintaining constructive interpersonal exchanges. Research on interpersonal interactions has demonstrated that emotional expressions serve as important social signals that influence individuals' perceptions of others and contribute to trust, rapport, and relationship quality Lee and Johnson (2025); Ueda and Yoshikawa (2022). Similarly, in educational contexts, teachers' emotional expressions influence classroom interactions and students' perceptions of teachers (Burić and Frenzel, 2023; Frenzel et al., 2021).

Second, teachers' emotions can directly influence students' emotions (Bieg et al., 2022; Frenzel et al., 2021; Wang et al., 2026). Even when teachers try to hide their emotions, students can often detect and match these emotions (Wang and Buric, 2023). Previous research indicates that both teachers' and students' emotions can be shared, both unintentionally and intentionally (Bieg et al., 2022; Frenzel et al., 2018). For instance, Wang et al. (2026) demonstrated that teachers' emotions could be transmitted to students through emotional transmission processes, whereby students' perceptions of teachers' enthusiasm shaped their own emotional experiences. Moreover, a longitudinal study by Frenzel et al. (2018) delved into the reciprocal relationship between teachers' and students' positive emotions, like enjoyment, within the classroom setting. It was found that when teachers demonstrated a sense of enjoyment at the start of the term, this had a positive impact on students' enjoyment by midterm. Similarly, when students showed enjoyment at the start of the term, it positively affected their teachers' enjoyment by midterm, highlighting a mutual exchange of positive emotions.

Third, teachers' emotions can significantly influence students' learning motivation (Ruzek et al., 2016). According to a cognitive-motivational model on the effects of emotions, our emotional responses play a crucial role in managing significant events. Emotions energize us both emotionally and physically, improve our focus, shape our thinking, and motivate us to take actions (Pekrun et al., 2002). Positive emotions (e.g., joy of learning or feeling supported by a teacher) may enhance motivation, whereas negative deactivating emotions (e.g., hopelessness, boredom) may reduce motivation (Frenzel et al., 2021; Mendzheritskaya et al., 2025). As motivation is crucial for student learning (Pekrun et al., 2002), the ability of emotions to evoke motivation may serve as an important mediating mechanism in the impact of teacher emotions on student outcomes. Frenzel et al. (2021) synthesized existing evidence suggesting that teachers' emotional experiences and expressions can shape students' motivational processes by influencing students' perceptions of the classroom environment, emotional experiences, and engagement in learning.

Despite the reliance on traditional methods to highlight the impact of teachers' emotions on student learning performance, there remains a notable gap in direct empirical evidence in the current literature. Students retrospectively report on their interpersonal relationships with teachers and their perceptions of the quality of instruction. Previous studies have shown that teachers' positive emotions enhance the quality of their interactions with students and elicit positive emotions and greater motivation in students (Frenzel et al., 2021; Lazarides et al., 2021; Xie and Derakhshan, 2021; Wang et al., 2026). Additionally, positive emotions in teachers, like enthusiasm and caring, have been proven to boost student engagement (Wang et al., 2026). This includes heightened attention to learning materials, increased cooperation, and better adherence to lessons, all of which are essential for active and meaningful learning (Gaspard and Lauermann, 2021; Lavy and Naama-Ghanayim, 2020).

However, most educational activities and materials are developed under the assumption that they cater to learners with low prior knowledge. Substantial evidence has emerged suggesting that instructional strategies effective for students with low prior knowledge may be ineffective or even harmful for students with high prior knowledge (Pi et al., 2023a; Jiang et al., 2023). This phenomenon is known as the expertise reversal effect (Kalyuga, 2007; Sweller et al., 2003). For instance, a Near-Infrared Spectroscopy (fNIRS) study by Pi et al. (2023a) found that strategies aimed at promoting student motivation can enhance interpersonal brain synchronization (IBS) in the temporoparietal junction (TPJ) and prefrontal cortex (PFC), as well as improve regulation-related behavioral sequences in students with low prior knowledge during co-explaining activities. However, these strategies did not provide benefits for students with high prior knowledge. Previous research has suggested that IBS reflects interpersonal connection and social alignment during social interactions, serving as a neural indicator of interpersonal coordination and shared cognitive processing (Li et al., 2022). Furthermore, prior knowledge also moderates the effects of instructional design on student learning performance (Jiang et al., 2023). For example, instructional supports that provide substantial guidance may facilitate learning among novices by compensating for their limited knowledge structures, whereas the same supports may hinder learning among knowledgeable learners by inducing redundancy effects.

Taken together, previous research suggests that teachers' emotions may influence student learning through multiple interconnected mechanisms. First, teachers' emotional expressions serve as important interpersonal signals that shape the quality of teacher–student interactions, fostering social connection and relational closeness (Burić and Frenzel, 2023; Frenzel et al., 2021; Holzer et al., 2024; Xie and Derakhshan, 2021). Second, teachers' emotions can be transmitted to students through emotional contagion processes, thereby influencing students' own emotional experiences (Bieg et al., 2022; Frenzel et al., 2021; Wang et al., 2026). Third, teachers' emotions may affect students' motivational processes by enhancing positive emotional experiences and engagement in learning (Frenzel et al., 2021; Ruzek et al., 2016). However, despite growing evidence that teachers' emotional expressions play an important role in students' interpersonal experiences, emotional states, and motivational processes, it remains unclear whether the effects of teachers' emotional expressions vary as a function of students' prior knowledge. Given that learners with different levels of prior knowledge possess different cognitive resources and may differ in their reliance on external social and emotional support (Li et al., 2022; Pi et al., 2023a), investigating the moderating role of prior knowledge is essential for understanding when and for whom teachers' emotional expressions are most beneficial.

In this study, we examined whether students' prior knowledge moderates the impact of pre-service teacher emotions on students' learning performance, interpersonal interactions evidenced by IBS in the frontal and temporal regions, and students' emotions and motivation during face-to-face instruction. We composed dyads consisting of a pre-service teacher and a student, with the pre-service teacher displaying either positive or neutral emotions. We hypothesized that pre-service teachers' positive emotions would enhance students' learning performance, the interpersonal interaction between teachers and students, as well as students' emotions and motivation. We expect the effects of pre-service teachers' positive emotions on students' learning performance, interpersonal interaction, emotions, and motivation to be stronger among students with low prior knowledge than among those with high prior knowledge.

## 2 Method

### 2.1 Participants and design

A sample of 74 undergraduate and graduate students from a Chinese university participated in this study and were organized into 37 teacher-student pairs (64 females, *M*_age_ = 20.69 ± 3.03 years). Among these participants, 37 were pre-service English teachers (i.e., English education majors) who served as teachers, while the remaining participants were from other majors and served as students. The pre-service teachers were selected because the instructional task involved teaching English vocabulary, which required participants with appropriate English language proficiency and pedagogical preparation. As part of their teacher education programmes, these participants had completed coursework and training related to English teaching and instructional practices. Teachers and students were matched by gender. Prior to this study, individuals in each dyad were not acquainted with one another. Informed consent was obtained from each participant before their participation in the experiment. The ethics committee of the first author's university approved the study protocols.

The experiment used a within-subject design. To determine whether the sample size was adequate, a *post-hoc* power analysis was conducted using G^*^Power 3.1 (Faul et al., 2007). The analysis indicated a statistical power of 0.84 (Cohen's *d* = 0.50), demonstrating that the sample size was sufficient to reveal the main effect of pre-service teacher emotion on the dependent variables in the within-subject design. All participants underwent two conditions in random order: teacher positive emotions vs. teacher neutral emotions. To minimize the effect of sequence on the results, 18 student participants first experienced instruction with a pre-service teacher's positive emotions, followed by a pre-service teacher's neutral emotions. Conversely, the other 19 student participants received instruction first with a pre-service teacher's neutral emotions and then with positive emotions. Data collection was conducted during the second semester of the 2023–2024 academic year.

### 2.2 Instruction task

In the experiment, pre-service teachers and students were seated in front of a computer to engage in face-to-face instruction using slides. Teacher participants employed either positive or neutral emotions to teach sets of English vocabulary words, with each teaching condition lasting approximately 4–5 min. The instruction covered English vocabulary words, their definitions, and example sentences, featuring seven words for each condition. Both undergraduate and graduate students are unfamiliar with the words used in the teaching material, showing no significant difference in their level of familiarity with these words (Pi et al., 2023b). The teaching materials consisted of emotionally neutral words that appear in international English language testing (IELTS), ensuring no significant difference in difficulty between the two conditions. Students were expected to learn these words and complete follow-up tests.

### 2.3 fNIRS data collection and analysis

#### 2.3.1 fNIRS data collection

We used LABNIRS (Shimadzu Corporation, Japan) to simultaneously record the levels of oxyhaemoglobin (HbO) and deoxyhaemoglobin (HbR) in paired participants of each dyad. The sampling rate was set at 10 Hz. We assigned 30 optodes (14 sources, 16 detectors) for each dyad. Each participant was assigned with 15 optodes (seven sources, eight detectors), forming 19 channels that covered the frontal and temporal regions.

**Figure 1:**
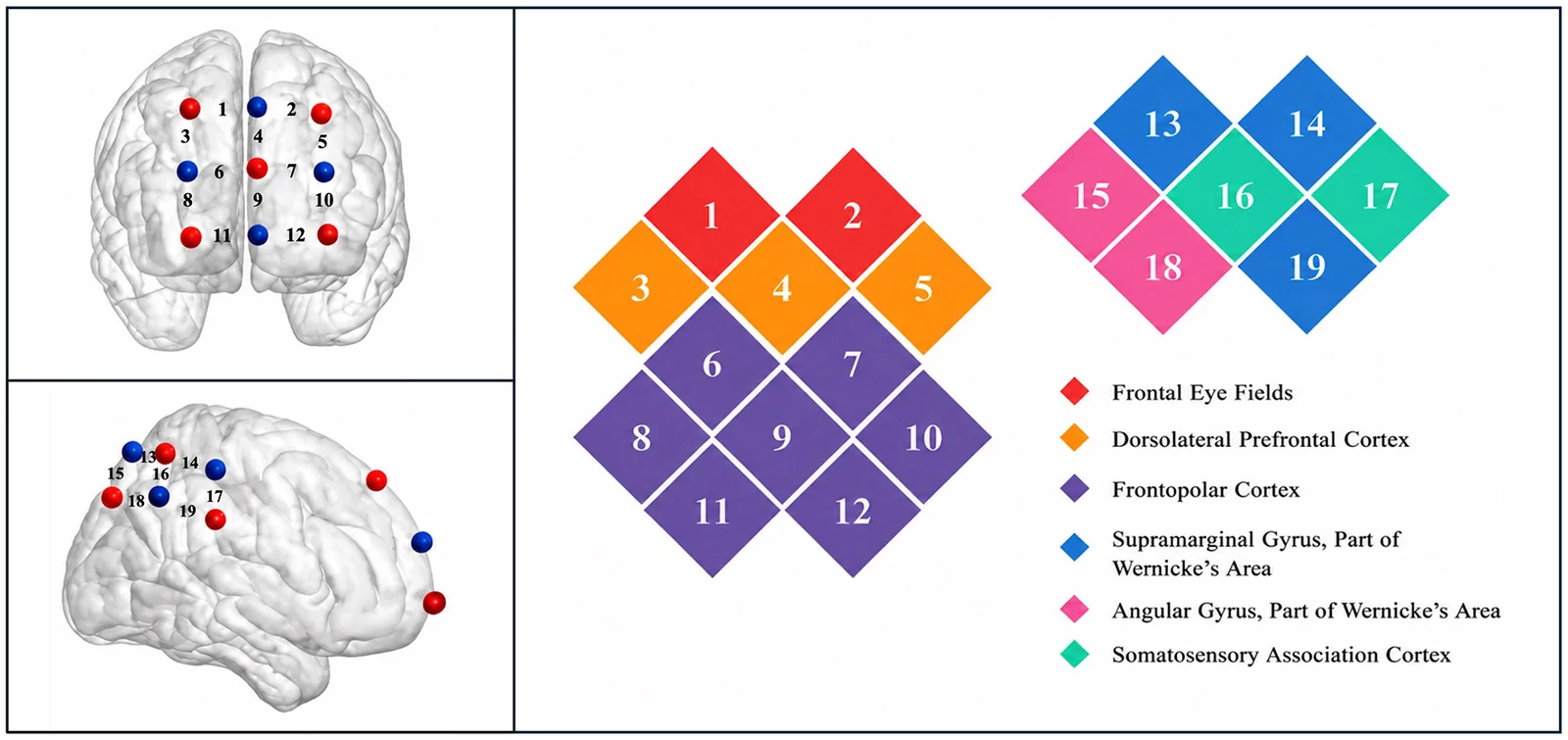
Optode probe set and channel layout. The red points represent sources, the blue points represent detectors, and the black numbers indicate brain channels.

In the study, optodes were positioned according to the international 10–20 system. All participants wore caps fitted with optodes, with a 3 × 3 optode probe set (four sources, five detectors) over the frontal cortex and a 2 × 3 optode probe set (three sources, three detectors) over the right temporoparietal junction. The spacing between each source and detector in the channels was 30 mm. Figure 1 illustrates the positions of the 19-channel optodes.

#### 2.3.2 fNIRS data analysis

**Figure 2:**
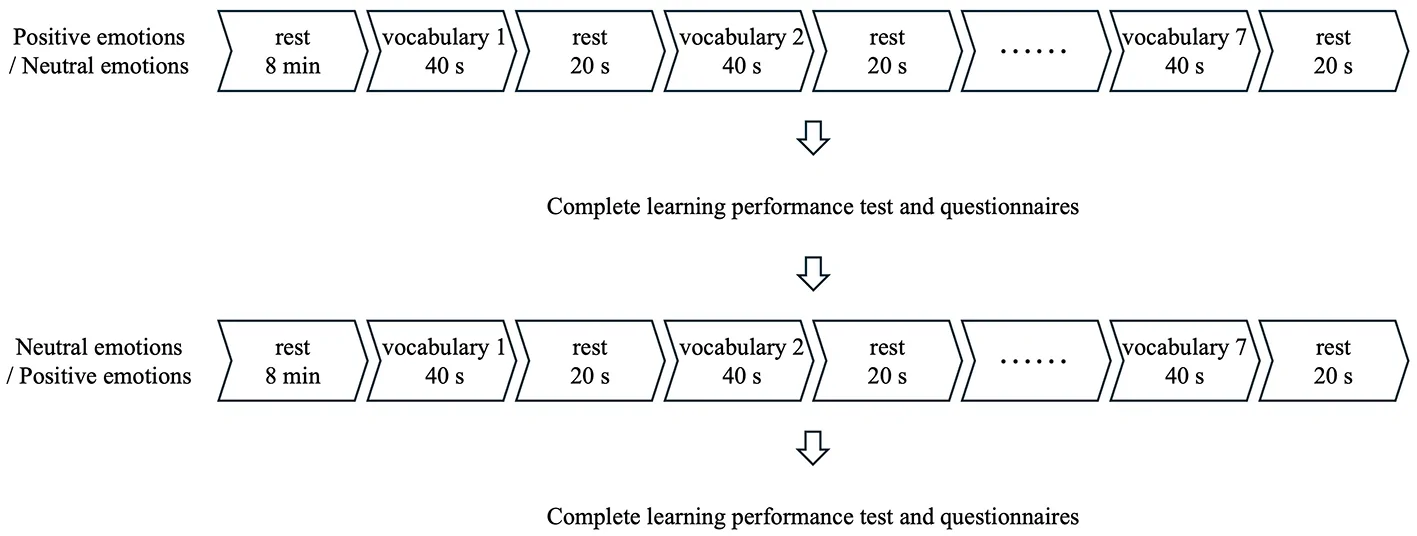
The experimental flowchart.

In fNIRS data analysis, Inter-Brain Synchronization (IBS) is used as a neural indicator of teacher-student interaction, representing the coherence of brain activity between pre-service teachers and students. First, given that the characteristics that the oxygenated hemoglobin signal (HbO) is more sensitive to changes in cerebral blood flow (Pan et al., 2017), we used the HbO signal for further analysis. To mitigate the impact of signal frequencies such as respiration (0.2–0.3 Hz) and heartbeat (0.7–4 Hz), we selected the frequency band of 6.25–33.33s (corresponding to 0.03–0.16 Hz) for subsequent analysis. This time window captured teacher-student interaction while excluding the effects of high and low-frequency noise on brain activity. As NIRS data represent relative values, we performed a Fisher z-statistics transformation on the coherence values. Second, we established regions of interest (ROIs) using the automatic anatomical labeling (AAL) method, based on the MNI coordinates of each channel (Pi et al., 2023a). There were six ROIs (Figure 2): frontal eye fields (SMA; channels 1 and 2), dorsolateral prefrontal cortex (dlPFC; channels 3–5), frontopolar cortex (FPC; channels 6–12), supramarginal gyrus (SMG; channels 13 and 14), Angular Gyrus (AG; channels 15 and 18), and somatosensory association cortex (SAC; channels 16 and 17). Third, we used Wavelet Transform Coherence (WTC) to assess the similarity between the NIRS signals of the teacher and the student in each dyad. This analysis allowed us to evaluate the degree of alignment in brain activity patterns between pre-service teachers and students during instruction. We performed WTC analysis for each channel and the rest of the ROIs on the converted HbO time series (19 pairs; Cui et al., 2012). Subsequently, we averaged the IBS between the same channel pairings. For example, we averaged the IBS value of channel 1 (a pre-service teacher) and channel 1 (a student) with the IBS value in each condition. This resulted in a total of 19 IBS pairings. Given that the IBS analyses were conducted on predefined ROIs related to teacher–student interaction rather than exploratory whole-brain analyses, subsequent moderation analyses were restricted to channel pairings showing significant learning-related IBS changes.

### 2.4 Measures

#### 2.4.1 Prior knowledge

Two researchers, experienced in teaching English and conducting research on language teaching, developed the prior knowledge test to assess students' English proficiency. The prior knowledge test comprises 21 fill-in-the-blank items, where participants were required to provide the Chinese meanings for each English word. An example item is: “Overhaul ().” Each correctly filled blank was valued at one point. All items were sourced from the IELTS vocabulary list. A higher total score indicates a greater level of participants' prior knowledge of English vocabulary. Cronbach's α in the current study was 0.95.

#### 2.4.2 Learning performance

The same two researchers developed the learning performance test, which includes seven single-choice questions. Participants were required to select the correct option from four choices. Each correct answer was valued at one point, allowing for a maximum possible score of seven. An example of the test is that: “Crack is a highly () and addictive derivative of cocaine. A. Juvenile; B. harass; C. Eccentric; D. potent.” Cronbach's α was 0.76.

#### 2.4.3 Emotion

We used the Self-Assessment Manikin (SAM) to measure students' perception of pre-service teachers' emotions and their own emotions. The SAM is a non-verbal test that requires participants to choose the cartoon figure that best represents their emotional state from a group of cartoon figures with different emotional states. The SAM includes two dimensions: (1) affective valence, which is the level of emotional pleasure, and (2) arousal, which is the level of emotional intensity (Bradley and Lang, 1994).

For the affective valence dimension, the question posed is: “How much pleasure are you currently experiencing? The figures below represent pleasure, ranging from ‘very unpleasant' on the left to ‘very pleasant' on the right. Each figure is associated with a number; please choose the number that best represents your emotional state.” For the arousal dimension, the question is: “how much calmness are you currently experiencing? The figures below represent levels of intensity, ranging from ‘very calm' on the left to ‘very intense' on the right. Each figure is associated with a number; please choose the number that best represents your emotional state.” The SAM employs a 9-point Likert scale, with higher scores indicating greater emotional valence.

#### 2.4.4 Motivation

We used the motivation dimension from the Learning Experience Questionnaire (Stull et al., 2018) to assess participants' motivation. The motivation dimension consists of six items designed to assess participants' enjoyment, willingness to learn, comprehension of the learning materials, desire to learn more about the subject, perceived usefulness of the lesson, and efforts to understand the material. An example of the items is as follows: “I feel motivated to understand the learning material.” The questionnaire utilizes a 7-point Likert scale, ranging from 1 (strongly disagree) to 7 (strongly agree). A higher score on the questionnaire reflects a higher level of participant motivation. The Cronbach's α was 0.96.

### 2.5 Procedure

Before the formal experiment, pre-service teacher participants underwent standardized pre-training during which they practiced delivering the instructional materials under both positive and neutral emotional conditions. The purpose of the training was to ensure consistency in instructional delivery and emotional expression across the two experimental conditions while minimizing variability unrelated to the experimental manipulation. One week after the training, teacher participants were evaluated on several criteria, including emotional expression (e.g., facial expressions, eyebrow movements, and speaking style), teaching intensity, speech rate, and the accuracy of the teaching materials. These evaluations were conducted to ensure the consistency of teachers' behavioral expressions and instructional delivery across conditions rather than to assess their internal emotional experiences. They had to meet the predefined criteria regarding the consistency of emotional expression, teaching intensity, speech rate, and instructional accuracy before proceeding to the formal experiment.

During the formal experiment, the dyads first filled out the demographic information, followed by the student participants filling out the prior knowledge test. Subsequently, the teacher-student pairs sat face-to-face and wore optodes caps. The experimental procedure was structured as follows: (1) in the resting phase, both participants relaxed for 8 min to record their baseline NIRS data; (2) in the instruction phase, the teacher participants taught two sets of words, and the student participants learned these words. Each word was taught for 15–30 s, followed by a 20-s rest after each instruction session. (3) upon completing each set of word teaching, the student participants completed the learning performance test, SAM, and motivation questionnaire in sequence. Experimental procedure was programmed using E-prime 2.0. Figure 2 shows the experimental process.

## 3 Results

Table 1 shows descriptive statistics for students' learning performance, IBS, emotions, and motivation in both the positive and neutral emotional expressions conditions. First, we conducted a series of paired-sample *t*-tests to check the effectiveness of manipulating pre-service teachers' emotional expressions (i.e., valence and arousal). Following this, we tested the moderation effects of students' prior knowledge using the Johnson-Neyman technique to identify the regions of significance (Carden et al., 2017). In all our analyses, we used pre-service teachers' emotional expressions as an independent variable and the score from the prior knowledge test as a moderation variable. We used the scores from the learning performance test, IBS, and students' emotions and motivation scales as dependent variables. In cases where no significant regions were identified, we performed a paired sample *t*-test to compare differences between the two conditions, thereby revealing the impact of pre-service teachers' emotional expressions.

**Table 1:** Descriptive statistics of students' learning performance, emotions, and motivation in the positive and neutral emotions conditions.

Dependent variable	Positive emotions		Neutral emotions	
	*M*	*SD*	*M*	*SD*
Learning performance	5.82	1.54	4.74	1.71
Students' emotion				
Valence	6.23	0.99	5.56	1.60
Arousal	4.15	1.48	3.85	1.48
Motivation	4.82	1.17	4.50	1.26

### 3.1 Manipulation check

We compared students' ratings of pre-service teachers' emotional expressions between the positive and neutral emotional expression conditions. The *t*-tests showed that the students reported higher scores when pre-service teachers displayed positive emotional expressions compared to when they displayed neutral emotions on emotional valence and arousal scales, *t*(36) = 3.44, *p* = 0.001; *t*(36) = 2.87, *p* = 0.007. These results confirmed the effectiveness of the manipulation of pre-service teachers' emotional expressions.

### 3.2 Learning performance

**Figure 3:**
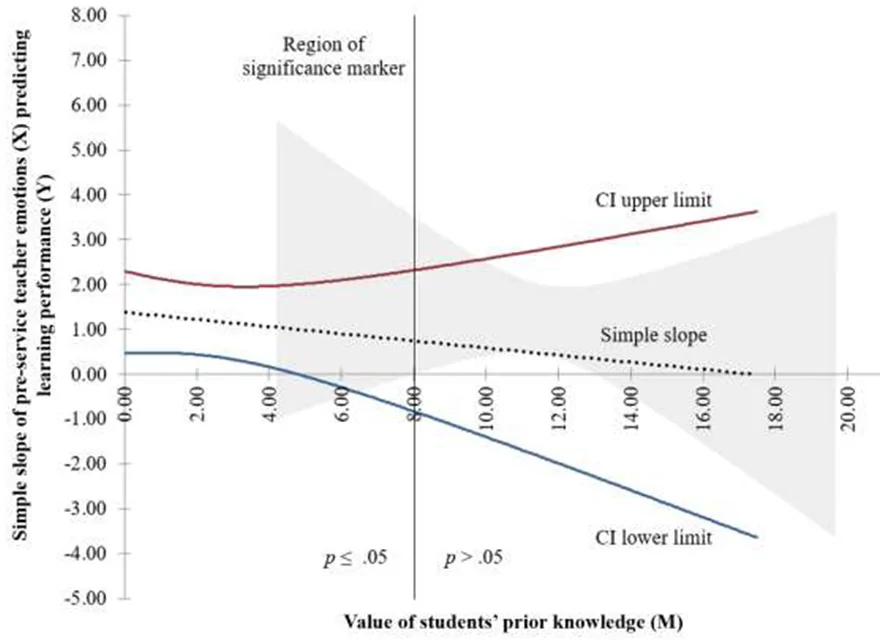
The results of floodlight analysis on learning performance. The effect of pre-service teachers' emotions on learning performance is significant when students' prior knowledge levels are ≤ 8.03.

The Johnson-Neyman floodlight analysis identified a significant region; the region of significance marker was 8.03 (1.07 *SD* above the mean, 8.11% of participants above). The results of the floodlight analysis are shown in Figure 3. Consistent with our hypothesis, participants with lower prior knowledge showed better learning performance in positive emotions conditions compared to the neutral emotions conditions. In contrast, participants with higher prior knowledge did not show a significant difference in learning performance between the two conditions.

### 3.3 Interpersonal brain synchronization

To examine if students' Inter-Brain Synchronization (IBS) while learning English vocabulary words differed from their baseline, we conducted a series of paired-sample *t-*tests to compare the differences between these two phases on the IBS values across all ROI pairings. When pre-service teachers used positive emotions, the IBS values of most ROI pairings in the learning phase were significantly higher than those in the rest phase (*p*s < 0.050), except for channel 1 (*p* = 0.178), channel 11 (*p* = 0.281), and channel 12 (*p* = 0.104). Furthermore, when pre-service teachers used neutral emotions, the IBS values of most ROI pairings in the learning phase were significantly higher than that in the rest phase (*p*s < 0.050), except for channel 1 (*p* = 0.215), channel 3 (*p* = 0.091), channel 8 (*p* = 0.063), channel 10 (*p* = 0.197), channel 14 (*p* = 0.092), and channel 17 (*p* = 0.241).

Subsequently, we explored the moderation effects of students' prior knowledge on the IBS of ROI pairings, for which the value in the learning phase was significantly greater than in the rest phase. The IBS values represented the differences between the learning and the rest phases. The Johnson-Neyman floodlight analysis identified significant regions which were channel 5 (dlPFC), channel 7 (Frontopolar cortex), channel 17 (SAC), and channel 18 (AG).

**Figure 4:**
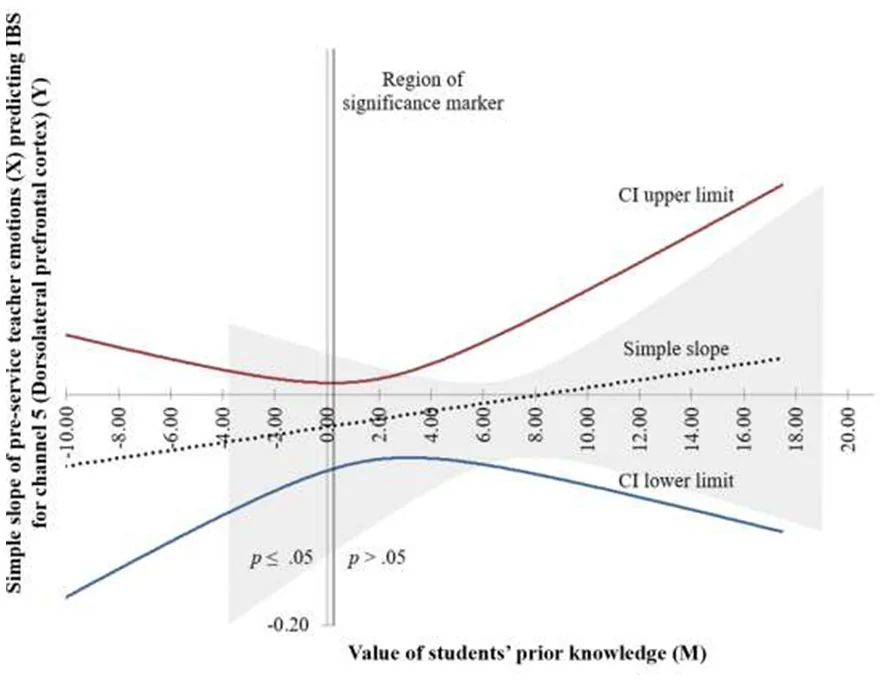
The results of floodlight analysis on IBS value for channel 5 (dlPFC). The effect of pre-service teacher emotion on IBS value for channel 5 is significant when students' prior knowledge levels are ≥0.27.

Regarding channel 5, the Johnson-Neyman floodlight analysis showed a significant region; the region of significance marker was 0.2682 (0.50 *SD* below the mean, 56.76% of participants below; Figure 4). Inconsistent with our hypothesis, participants with lower prior knowledge exhibited significantly lower IBS in positive emotions conditions compared to the neutral emotions conditions. However, participants with higher prior knowledge did not differ significantly between the two conditions.

**Figure 5:**
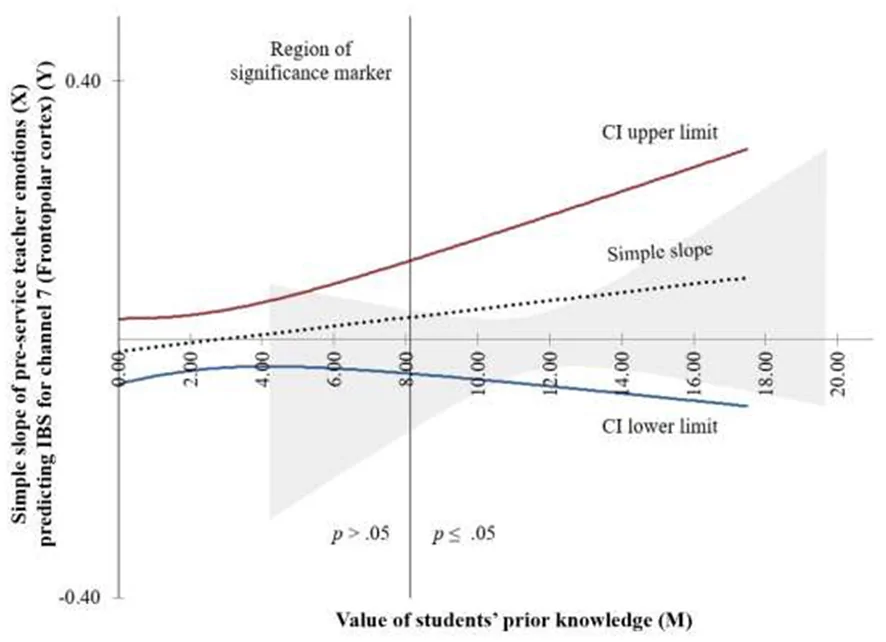
The results of floodlight analysis on IBS value for channel 7 (Frontopolar cortex). The effect of pre-service teacher emotion on IBS value for channel 7 is significant when students' prior knowledge levels are ≥8.11.

Regarding channel 7, the Johnson-Neyman floodlight analysis showed a significant region; the region of significance marker was 8.11 (1.09 *SD* above the mean, 8.11% of participants above; Figure 5). Inconsistent with our hypothesis, participants with lower prior knowledge did not exhibit a significant difference between the two conditions. However, participants with higher prior knowledge showed greater IBS in the positive emotions conditions compared to the neutral emotions conditions.

**Figure 6:**
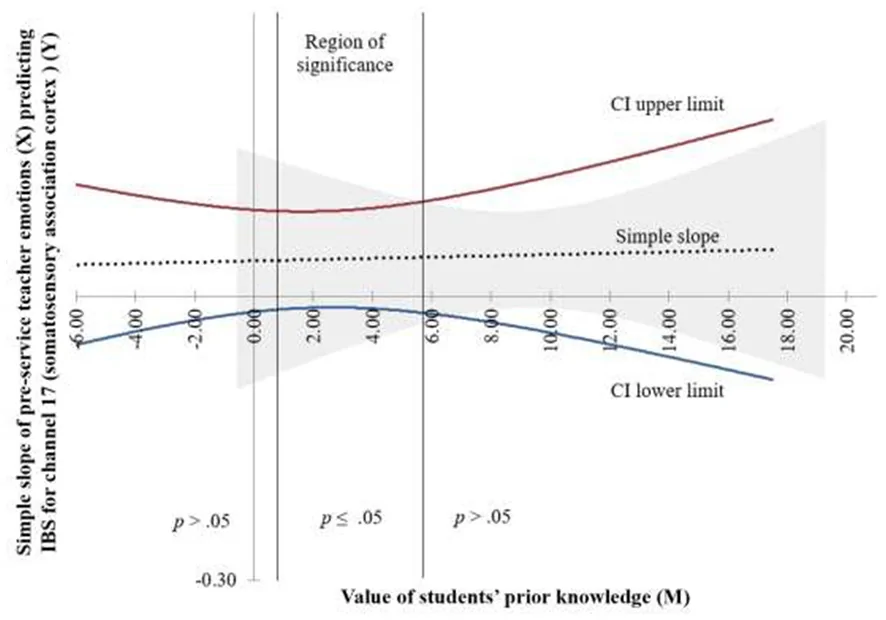
The results of floodlight analysis on IBS value for channel 17 (SAC). The effect of pre-service teacher emotion on IBS value for channel 17 is significant when students' prior knowledge levels fall between 0.79 and 5.71.

Regarding channel 17, the Johnson–Neyman floodlight analysis also showed a significant region with two markers of significance (Figure 6). The first marker was at 0.79 (0.39 *SD* below the mean, 56.76% of participants below), and the second was at 5.71 (0.60 *SD* above the mean, 56.76% above). Consistent with our expectations, participants with prior knowledge levels between 0.79 and 5.71 exhibited greater IBS in the positive emotions conditions compared to the neutral emotions conditions. However, participants with prior knowledge levels below 0.79 or above 5.71 did not exhibit a significant difference in IBS between the two conditions.

**Figure 7:**
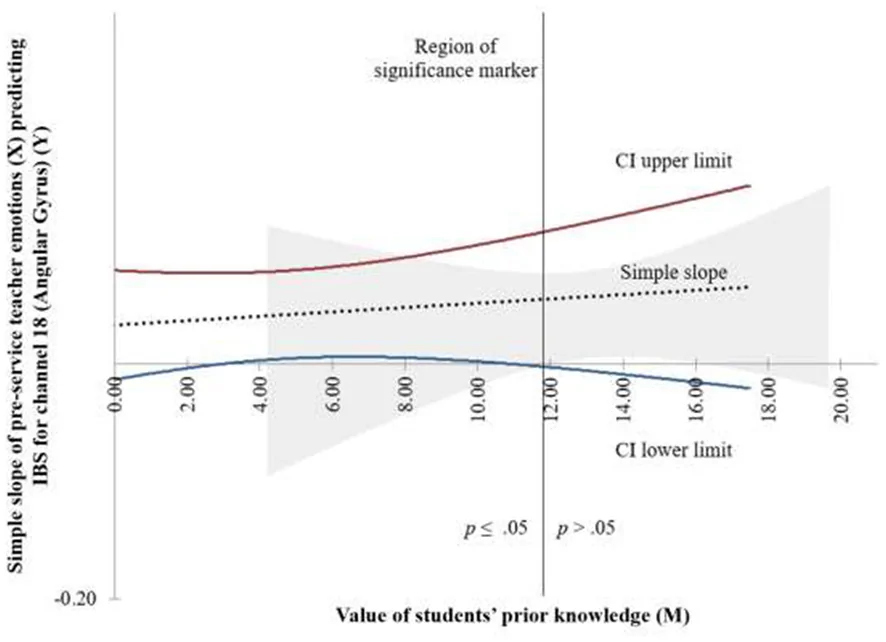
The results of floodlight analysis on IBS value for channel 18 (AG). The effect of pre-service teacher emotion on IBS value for channel 18 is significant when students' prior knowledge levels are ≤ 11.80.

Regarding channel 18, the Johnson-Neyman floodlight analysis also showed a significant region; the region-of-significance marker was at 11.80 (1.83 *SD* above the mean, 5.41% of participants above; Figure 7). Consistent with our hypothesis, participants with lower prior knowledge exhibited greater IBS in the positive emotions conditions compared to the neutral emotions conditions. In contrast, participants with higher prior knowledge did not show a significant difference between the two conditions.

### 3.4 Students' emotions and motivation

**Figure 8:**
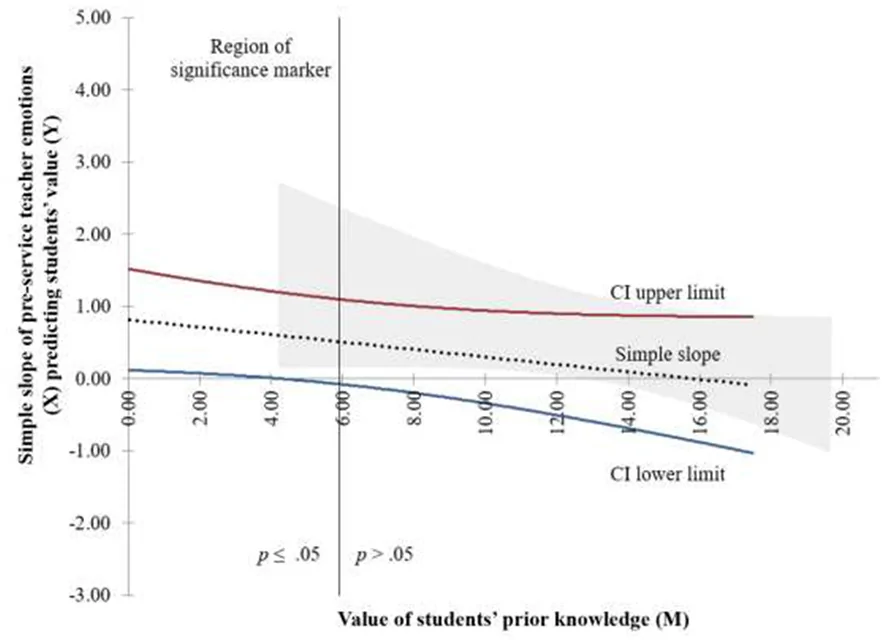
The results of floodlight analysis on students' valence. The effect of pre-service teacher emotions on students' valence is significant when students' prior knowledge levels are ≤ 5.92.

Regarding students' valence, the Johnson-Neyman floodlight analysis showed a significant region; the region of significance marker was at 5.92 (0.64 *SD* above the mean, 13.51% of participants above; Figure 8). Consistent with our hypothesis, participants with lower prior knowledge (i.e., those who were *SD* below the mean) reported more positive emotions in the positive emotions condition than in neutral emotions condition. In contrast, participants with higher prior knowledge did not show a significant difference between the two conditions. However, regarding students' arousal, the Johnson-Neyman floodlight analysis did not show any significant region. Similarly, a paired sample *t*-test found no significant difference between the two conditions (*t*(36) = 1.87, *p* = 0.070).

**Figure 9:**
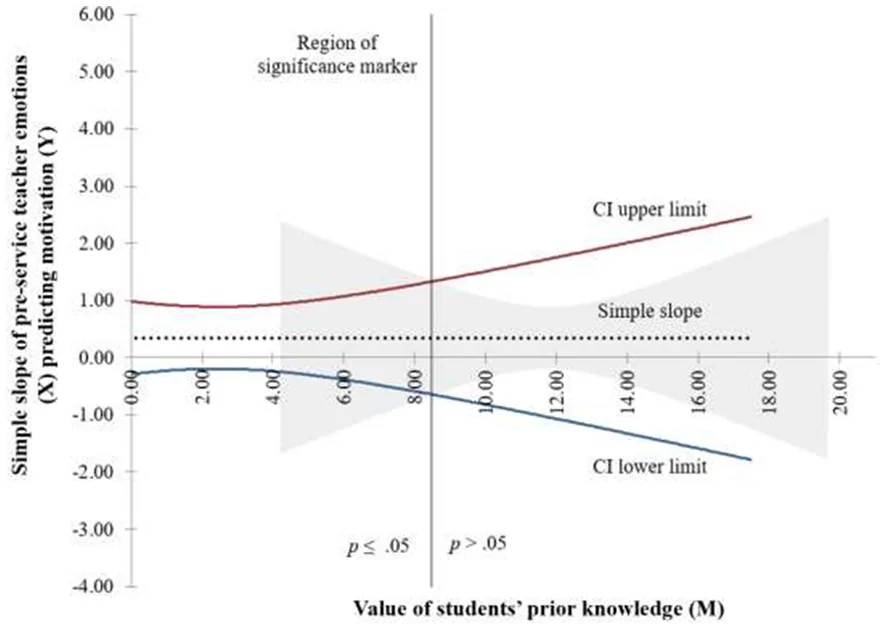
The results of floodlight analysis on students' motivation. The effect of pre-service teacher emotions on motivation is significant when students' prior knowledge levels are ≤ 8.47.

Regarding students' motivation, the Johnson–Neyman floodlight analysis showed a significant region; the region of significance marker was at 8.47 (1.16 *SD* above the mean, 8.11% of participants above; Figure 9). Consistent with our hypothesis, participants with lower prior knowledge showed greater motivation in the positive emotions conditions compared to the neutral emotions conditions. However, participants with higher prior knowledge did not show a significant difference between the two conditions.

## 4 Discussion

The current study provides direct evidence of the effect of pre-service teachers' positive emotional expressions and the moderating effect of students' prior knowledge on students' learning performance, IBS, emotions, and motivation. Consistent with our hypothesis, we found that students with relatively low prior knowledge exhibited better learning performance when taught by pre-service teachers who displayed positive emotions, compared to those who were neutral. The results align with the expertise reversal effect, which suggests that instructional design elements (e.g., visual cues) are effective for students with low prior knowledge but can negatively impact those with high prior knowledge (Kalyuga, 2007; Sweller et al., 2003). One possible explanation is that learners with low prior knowledge possess fewer well-developed knowledge structures and therefore rely more heavily on external instructional support during learning (Jiang et al., 2023). In face-to-face instruction, teachers' positive emotional expressions may increase students' willingness to engage in communication and encourage sustained attention to instructional content (Burić and Frenzel, 2023; Frenzel et al., 2021; Holzer et al., 2024; Xie and Derakhshan, 2021). These supportive expressions may compensate for students' limited internal cognitive resources, thereby facilitating learning. In contrast, learners with high prior knowledge possess richer domain-specific schemas and can regulate their own learning more effectively. Consequently, they rely less on external emotional support, resulting in a diminished benefit of pre-service teachers' positive emotional expressions.

The findings concerning IBS, as well as students' emotions and motivation in this study, further support the expertise reversal effect. Specifically, we found that students with high prior knowledge showed greater IBS in the FPC region in response to positive emotions from teachers, compared to neutral emotions. In contrast, students with low prior knowledge showed an opposite response in the dlPFC region when pre-service teachers showed positive emotions, as opposed to neutral ones. Prior research has indicated that increased IBS in the FPC and dlPFC regions as a neural marker for social cognitive activities, such as emotional processing, theory of mind, perspective-taking, and attention (Gvirts and Perlmutter, 2020; Li et al., 2022; Sun et al., 2020). Therefore, one possible interpretation is that the greater IBS observed in the FPC region reflects enhanced interpersonal coordination during the processing of teachers' emotional expressions. However, the specific cognitive processes underlying this synchronization were not directly examined in the present study. Notably, although students with high prior knowledge exhibited greater IBS in the FPC region, this neural synchronization did not correspond to improved learning performance.

Further support for the moderating role of prior knowledge comes from the IBS findings in the temporal regions (i.e., SAC and AG). We found that students with low prior knowledge showed greater IBS in SAC and AG regions when pre-service teachers displayed positive emotions, as opposed to neutral ones. In contrast, students with high prior knowledge did not show a significant difference between the two emotion conditions. The SAC has been implicated in integrating information within and across sensory modalities (Dahl et al., 2015). In our study, students' learning involved processing slides on the screen, teachers' speech, and various emotional cues (e.g., raising eyebrows and corners of the mouth, vocal volume and speed) during face-to-face instructions. Consequently, students had to integrate multimodal information for effective learning. Furthermore, the AG has been associated with episodic and semantic cognition (Humphreys et al., 2021; Thakral et al., 2017). Therefore, positive teacher emotions were associated with greater IBS in the SAC and AG regions among learners with low prior knowledge. This increase may reflect more efficient interpersonal coordination during multimodal communication, although the specific behavioral processes underlying this synchronization require further investigation. Such interpersonal coordination may have provided additional support for integrating instructional content with pre-service teachers' emotional expressions, which could be particularly beneficial for learners with low prior knowledge who rely more heavily on external instructional support.

The self-report measures revealed a similar pattern. Specifically, students with relatively low prior knowledge reported more positive emotions and higher motivation when taught by pre-service teachers who displayed positive emotions, compared to neutral emotions. In contrast, students with high prior knowledge did not report changes in emotions or motivation, despite showing increased IBS in the frontal regions. This pattern further suggests that perceiving teachers' emotional expressions does not necessarily lead to emotional transmission or motivational benefits. Rather, these findings suggest that the effectiveness of teachers' positive emotional expressions may depend on learners' need for external emotional support, which may be greater among learners with low prior knowledge. This observation supports the expertise reversal effect in relation to students' self-reported emotions and motivation. It is worth noting that the differences observed between IBS in the FPC and dlPFC regions and self-reported emotions for students with relatively high prior knowledge, suggest that perceiving emotions does not guarantee their transmission. Our findings for students with low prior knowledge align with existing studies on the transmission of emotions between teachers and students (Bieg et al., 2022; Frenzel et al., 2021; Wang et al., 2026). Learners with low prior knowledge may depend more strongly on teachers' affective cues to interpret the learning context, making them more susceptible to emotional transmission than learners with high prior knowledge (Jiang et al., 2023). Together, these findings suggest that teachers' positive emotions may facilitate learning among students with low prior knowledge by promoting more adaptive interpersonal interactions, more positive emotional experiences, and higher learning motivation, all of which are associated with improved learning performance.

There are a few limitations that need to be addressed. First, the instructional context in this study was a brief, one-to-one experimental interaction involving pre-service teachers rather than experienced in-service teachers in authentic classroom settings. Although the pre-service teachers possessed relevant pedagogical knowledge and received standardized training before the experiment, their instructional behaviors and emotional expressions may differ from those of experienced teachers. Moreover, because teaching and learning were examined through a single short-term intervention, the findings may not fully generalize to longer-term teaching and learning processes. Therefore, caution should be exercised when extending the present findings to authentic classroom contexts or sustained instructional practices. Future research should replicate this study with experienced teachers, larger-scale classroom settings, and longitudinal designs to examine whether similar effects emerge over extended periods of instruction. In addition, the study was conducted in a Chinese university context. Given that the expression and interpretation of teachers' emotions may vary across cultural contexts, future research should examine whether similar patterns can be observed among learners and teachers from different educational and cultural backgrounds. Third, although the IBS analyses were conducted based on theoretically defined ROIs and task-related changes in brain synchronization, multiple comparisons remain a potential concern in neuroimaging analyses. Future studies with larger samples should apply more comprehensive correction approaches to further validate the robustness of IBS findings. Fourth, this study primarily focused on teachers' positive emotional expressions rather than directly assessing teachers' experienced emotional states. Although teachers' facial expressions, eyebrow movements, and speaking styles were evaluated before the experiment to ensure the effectiveness and consistency of emotional expression, future research should further distinguish between teachers' internal emotional experiences and their behavioral expressions. Finally, positive emotions represent a broad construct that may include different emotions (e.g., enthusiasm, enjoyment, and humor), which may differ in their likelihood of being perceived and transmitted by students (Bieg et al., 2022). Future research should examine whether different types of teachers' positive emotions produce distinct effects on students' learning and interpersonal experiences.

A critical finding of this study is that students with low prior knowledge benefited more from pre-service teachers' positive emotional expressions compared to students with high prior knowledge. Overall, these findings extend the expertise reversal effect to teachers' emotional expressions in face-to-face instruction by demonstrating that the effectiveness of positive teacher emotions depends on learners' prior knowledge. This highlights the importance of considering learner characteristics when designing emotionally supportive instructional practices. From a practical perspective, our findings suggest that positive emotional expressions by teachers may be particularly beneficial when supporting learners with relatively low prior knowledge. Such emotional expressions may facilitate interpersonal interaction, promote positive emotional experiences, and enhance learning motivation. However, given that the present study was conducted in a controlled one-to-one instructional setting using pre-service teachers, these practical implications should be interpreted with caution. Future studies involving experienced teachers and authentic classroom contexts are needed before broader educational recommendations can be made.

## Data Availability

The original contributions presented in the study are included in the article/supplementary material, further inquiries can be directed to the corresponding authors.
